# A theoretical review of the role of teacher professional development in EFL students’ learning achievement^☆^

**DOI:** 10.1016/j.heliyon.2023.e15806

**Published:** 2023-04-27

**Authors:** Jing Zeng

**Affiliations:** a Hubei University of Education, Wuhan, Hubei, 430205, China; b Teacher Education Research Center of Hubei, Wuhan, Hubei, 430205, China

**Keywords:** Learning achievement, Teacher professional development, EFL students, Theoretical review

## Abstract

Due to the value of teacher professional development in increasing student learning achievement, an increasing number of articles in general education have assessed the impact of this professional trait on student achievement. Nevertheless, in language education, a few investigations have examined the role of professional development in student learning achievement. Moreover, no inquiry has theoretically reviewed the implications of teacher professional development for EFL learners’ achievement. This theoretical review intends to address the gap by concentrating on the potential effects of teacher professional development on EFL students’ learning achievement. The empirical and theoretical evidence were thus inspected with a view to explaining the role of teacher professional development in English learners’ academic outcomes. Consequently, the prominent role of teacher professional development in improving EFL students’ learning achievement was proved using the relevant evidence. The present review’s outcomes may be useful and illuminating for teachers, teacher educators, and educational managers.

## 1 Introduction

The end of any educational program is to lead students to the best possible outcomes [1,2]. Simply put, promoting students’ learning achievement is an important goal that all educational programs have in common. Student learning achievement is “the outcome of learning, which is typically measured by classroom grades, classroom assessments, and external achievement tests” [ [3], p. 269]. Put differently, student learning achievement has to do with how well individual students have mastered the learning content [4,5]. Likewise, students’ learning achievement in language learning programs is concerned with the degree to which they have acquired the language learning materials [6,7]. Teachers, as the main source of knowledge in classroom contexts, are believed to play an essential role in promoting students’ learning achievement [8,9]. In fact, there is a consensus among academics that differences in students’ achievement levels can be attributed to their teachers’ characteristics [[10], [11], [12]]. In light of this notion that teachers can cause a significant difference in learners’ achievement levels, several researchers have analyzed the role of teachers’ personal (i.e., emotional intelligence, personal beliefs, self-efficacy, humor, etc.) and professional traits (i.e., rapport, clarity, confirmation, support, credibility, job satisfaction, teaching creativity, teaching styles, work engagement, etc.) in students’ learning achievement [e.g., Refs. [[13], [14], [15], [16], [17], [18], [19], [20], [21], [22], [23], [24], [25], [26], [27], [28], [29]], among others]. Teacher personal traits, also called personal resources, refer to the physical and emotional resources teachers bring with them to educational environments [12]. Teacher professional traits, on the other hand, pertain to those features or characteristics that teachers attain in educational environments [12]. Teacher professional development as an important instance of professional traits has also been the focus of numerous investigations.

As a profession related construct, professional development generally pertains to the growth of a person in his or her career [30,31]. For instructors, “professional development” is a necessity and an opportunity, acting as a forum for improvement and reinforcement of present instructional practice [32,33]. It is both individual and social in nature [34], and it has become a priority for all educators who wish to enhance learners’ academic achievements [35]. According to Avalos [36], teacher professional development refers to the professional growth teachers attain as a result of obtaining more experience and critically evaluating their instructional practices. Desimone and Stuckey [37] further defined this concept as “a necessary mechanism that can deepen teachers’ content knowledge and enhance their teaching practices” [p. 468]. This mechanism, according to Dunst, Bruder, and Hamby [38], will be more efficient if it is prolonged. As put forward by Sims and Fletcher-Wood [39], collaboration also makes this process more effective as “it gives teachers the chance to challenge each other and clarify misunderstandings” [p. 3].

As noted by Abou-Assali [40], teachers’ professional development is associated with improved student achievement. He maintained that teachers who engage in sustained professional development programs commonly demonstrate improvements in their instructional practices, which is critical for increased student achievement. In this regard, Meissel, Parr, and Timperley [41] also mentioned the professional growth that arises as teachers move along the vocational cycle can notably promote pupils’ learning attainment. Additionally, Hoge [42] stated that teachers who continually develop their subject knowledge, professional skills, and teaching abilities are able to lead their students towards improved learning achievement. Due to the prominent role of teacher professional development in raising student attainment [[40], [41], [42]], a growing number of inquiries in mainstream education have been devoted to the impacts of this construct on students’ learning achievement [e.g., Refs. [[43], [44], [45], [46], [47], [48], [49]], among others]. In language education, on the other hand, the effects of teacher professional development on student achievement have been rarely investigated [50,51]. Moreover, as the review of available literature revealed, no theoretical review has been done on the role of teacher professional development in EFL students’ learning achievement. Theoretical review is one of the outstanding research approaches that helps researchers review the theoretical backgrounds and empirical evidence of a particular topic. Guided by a theoretical review approach, the present study strives to explicate the role of teacher professional development in EFL students’ learning achievement.

### 1.1 EFL students’ learning achievement

Student learning achievement, also known as student attainment, generally means what individual students have accumulated throughout the learning process [52,53]. When it comes to language learning classes, student learning achievement pertains to “the overall attainment of students in a language course” [ [54], p. 950]. As put forward by Haines and Mueller [55], student learning achievement has to do with the accomplishment of course objectives by individual students. Since course objectives are commonly multifaceted [56], student learning achievement is thought to be a multidimensional concept that is commonly measured by course grades [57,58].

### 1.2 EFL teachers’ professional development

Teacher professional development has been primarily conceptualized by Glatthorn [59] as “the growth that occurs as the teacher moves through the professional career cycle” [p. 41]. This concept further defined as “a long term process that includes regular opportunities and experiences planned systematically to promote growth and development in the profession” [ [60], p. 12]. Teacher professional development, according to this definition, is an ongoing process in which teachers, as reflective practitioners, critically evaluate their subject knowledge, teaching skills, and instructional strategies and improve them over time [[61], [62], [63], [64]]. For Richter, Kunter, Klusmann, Lüdtke, and Baumert [65], teacher professional development pertains to various educational experiences related to the teaching profession that are intended to improve teaching practices and outcomes. According to them, these educational experiences may be formal (e.g., attending webinars, workshops, teacher training courses, etc.) or informal (e.g., reading scholarly articles, talking with colleagues, etc.). Formal professional development experiences happen when teachers engage in activities systematically designed by an educational committee [66,67]. These systematic activities are commonly designed with the aim of deepening and extending teachers’ content knowledge and instructional skills [68]. Informal professional development experiences can take on a variety of formats where teachers update their subject knowledge and teaching skills through reflection, reading scholarly articles, and talking with colleagues [69,70].

### 1.3 The role of teacher professional development in promoting EFL students’ learning achievement

To elucidate the consequences of teacher professional development for EFL students’ learning achievement, Borg [71] maintained that teacher professional development is linked to improved student achievement. To him, teachers who regularly engage in formal and informal experiences of professional development are more successful in raising their students’ learning attainments. It is mostly because such experiences bring about favorable changes in teachers’ subjective knowledge and instructional skills [72,73], which empower them to effectively improve their students’ achievement [74,75]. In a similar vein, Patton, Parker, and Tannehill [76] pinpointed the pivotal role of teacher professional development in students’ learning outcomes by mentioning the positive changes that professional development programs make in teaching practices. They articulated that professional development programs typically result in the acquisition of new instructional knowledge and skills, which inspire teachers to refine their teaching practices [77]. As they clearly explained, using effective teaching practices helps teachers enhance their pupils’ learning outcomes. Further, Kennedy [78] also argued that teacher professional development can make a huge difference in learners’ achievement levels. She believed that professional development experiences equip teachers with novel teaching strategies that are essential for promoting learners’ accomplishments. In this regard, King [79] postulated that professional development programs help instructors assess their pedagogical knowledge, reflect on their instructional practices, and improve the teaching quality. The higher the teaching quality, the greater the students’ learning outcomes [80,81]. Additionally, Earley and Porritt [82] also attempted to illustrate the value of teacher professional development in improving student achievement by referring to the central purpose of professional development programs. They stated that leading pupils to increased learning achievement is what teacher professional development programs are designed for. Given this, teachers who constantly take part in professional development programs can assist their learners to reach higher levels of learning achievement.

## 2 Empirical evidence

To date, in mainstream education, several research studies have been carried out to uncover the impacts of teacher professional development on student learning achievement [e.g., [[45], [48], [83], [84], [85], [86]], among others]. Althauser [83], for example, examined the influence of job-embedded professional development on math students’ achievement. To this aim, 35 instructors and students were selected from 10 elementary schools in United States. To measure students’ achievement in mathematics, the state mathematics content test was implemented. Then, to examine math teachers’ development in their profession, a self-report scale was employed. Finally, the outcomes of multiple regression indicated that math teachers’ professional development is a significant predictor of their students’ learning achievement. Put differently, students’ achievement in mathematics can be positively influenced by their instructors’ professional development. By the same token, Jacob et al. [45] evaluated the effects of professional development programs on teachers’ instructional knowledge and students’ learning achievement. For this purpose, 105 school teachers were invited to participate in this inquiry. Participants were divided into control and treatment groups at random. Those who were assigned to the experimental group were asked to attend the professional development program designed by the researchers. Finally, the results of the analyses revealed that the professional development program favorably affected teachers’ knowledge and students’ achievement as well. Moreover, Fischer et al. [84] investigated the implications of teacher professional development for learners’ achievement. To do so, two valid scales measuring teacher professional development and student learning achievement were given to a large sample of 133336 students and 7434 teachers. The multi-level structural equation models indicated that teacher professional development can noticeably affect student achievement. Furthermore, Lu et al. [48] explored the influence of teacher professional development on Chinese students’ achievement. To accomplish this, two self-report scales were administered to a group of Chinese teachers and students. Participants’ responses evinced that Chinese students’ achievement can be favorably affected by teacher professional development. In a same vein, Tanveer et al. [86] inspected the role of teacher professional development in students’ learning achievement. In doing so, two self-developed questionnaires were distributed among 170 college students and teachers. Using regression analyses, the scholars found that student achievement can be dramatically improved by teacher professional development.

Besides, in language education, a few investigations have also been conducted in this regard [[87], [88], [89]]. For one, Akiba and Liang [87] evaluated the impact of teacher professional development on language learners’ achievement. To accomplish this, two questionnaires were distributed among 467 teachers and 11192 learners. The data analysis demonstrated that teacher professional development can substantially affect pupils’ achievement. In their study, Van Kuijk et al. [89] measured the degree to which EFL students’ reading achievement can be improved through teacher professional development. To do this, the participants were randomly sorted into control and treatment groups. Finally, they discovered that those who were in the treatment group attained much more achievement. Likewise, Cantrell et al. [88] assessed the function of teacher professional development in language learners’ reading achievement. To accomplish this, 21 in-service teachers and their learners were selected to be assigned to treatment and control groups. Teachers in the treatment group took part in formal professional development programs. The results of independent sample *t*-test uncovered that learners whose teachers took part in the professional development programs outperformed their classmates. Simply said, learners in the treatment group attained higher levels of reading achievement than those in the control group. Given the limited attention that has been placed on the role of teacher professional development in students’ language achievements [[87], [88], [89]], future researchers are advised to investigate this topic in different language learning environments, including English classes.

## 3 Conclusion and pedagogical implications

This study was set out to elucidate the role of teacher professional development in improving EFL students’ learning achievement. Using existing evidence, EFL students’ learning achievement was proved to be favorably affected by teacher professional development. Accordingly, it is fair to conclude that those teachers who continually participate in professional development experiences can significantly improve students’ learning achievement. This seems to be extremely useful and insightful for teacher educators, teachers, and educational managers in any educational program. With respect to the present review’s finding regarding the prominence of teacher professional development in improving student achievement, teachers are advised to regularly engage in formal and informal professional development experiences. Participating in professional development programs enables in-service teachers to extend their subject knowledge and instructional skills [68]. This, in turn, empowers them to enhance their students’ academic outcomes [71,74,75]. With respect to the role of professional development programs in teaching quality, educational managers are expected to provide teachers with effective development programs to help them improve their subject knowledge and teaching skills. They are also required to increase teachers’ income in order to motivate them to continue their development throughout their professional lives. Additionally, due to the pivotal role that teacher professional development serves in improving students’ learning outcomes, teacher educators should educate their teacher students on how to develop in the teaching profession. In fact, they need to teach in-service instructors how to assess their pedagogical knowledge and reflect on their instructional practices, which are critical for their professional growth.

## Author contribution statement

All authors listed have significantly contributed to the development and the writing of this article.

## Data availability statement

No data was used for the research described in the article.

## Additional information

No additional information is available for this paper.
